# Treatment of candidemia and invasive candidiasis in the intensive care unit: *post hoc *analysis of a randomized, controlled trial comparing micafungin and liposomal amphotericin B

**DOI:** 10.1186/cc8117

**Published:** 2009-10-05

**Authors:** Bertrand F Dupont, Olivier Lortholary, Luis Ostrosky-Zeichner, Flavie Stucker, Vijay Yeldandi

**Affiliations:** 1 Université Paris Descartes, Hôpital Necker-Enfants Malades, Centre d'Infectiologie Necker-Pasteur, 149 rue de Sevres, 75015 Paris, France; 2 Centre National de Référence Mycologie et Antifongiques, Institut Pasteur (CNRS URA3012), 25 rue du Docteur Roux, 75724 Paris, France; 3 University of Texas, 6431 Fannin St, John Freeman Building, Houston, TX 77030, USA; 4 Astellas Pharma BV, Elisabethhof 19, 2353 EW Leiderdorp, The Netherlands; 5 Westlake Hospital, 1111 Superior Street, SUITE 101, Melrose Park, IL 60160, USA

## Abstract

**Introduction:**

Invasive candidiasis and candidemia are life-threatening nosocomial infections in intensive care patients.

**Methods:**

A *post hoc *analysis of a phase 3 trial assessing micafungin (100 mg/day for subjects > 40 kg; 2 mg/kg/day for subjects ≤ 40 kg) versus liposomal amphotericin B (3 mg/kg/day). Subgroups were defined according to the type of ward on the first day of treatment: intensive care unit (ICU) or non-ICU. Multivariate regression was performed to identify factors associated with treatment success at end of therapy and all-cause mortality at days 8 and 30.

**Results:**

In non-ICU subjects, treatment success was significantly higher for micafungin versus liposomal amphotericin B (85% (n = 108/127) versus 72.1% (n = 98/136); *P *= 0.0113). However, for ICU subjects, treatment success rates for micafungin versus liposomal amphotericin B were similar (62.5% (n = 75/120) versus 66.4% (n = 73/110); *P *= 0.5828). Overall, treatment success was significantly lower in ICU subjects compared with non-ICU subjects (64.3% (n = 148/230) versus 78.3% (n = 206/263); *P *= 0.0006). Multivariate regression analysis revealed a lower likelihood of treatment success for: ICU versus non-ICU subjects; persistent neutropenia; and high versus low Acute Physiology and Chronic Health Evaluation (APACHE) II scores. However, when interactions between potential explanatory factors were included in the analysis model, ICU status no longer emerged as a significant associated variable but the association between APACHE II score and treatment outcome remained. Further analyses indicated that the likelihood of mortality at day 8 and day 30 was lower for subjects with lower APACHE II scores. Renal function was significantly better in micafungin versus liposomal amphotericin B subjects: a difference (liposomal amphotericin B - micafungin in mean peak change in estimated glomerular filtration rate (ml/minute/1.73 m^2^) of -18.2 (*P *< 0.0001) and -17.7 (*P *= 0.0124) in non-ICU and ICU subjects, respectively.

**Conclusions:**

Overall, ICU subjects had lower treatment success rates than non-ICU subjects for both liposomal amphotericin B and micafungin. Multivariate regression after controlling for potential confounding factors suggested the APACHE II score remained a potential explanatory factor associated with treatment success, mortality at day 8, and mortality at day 30.

**Trial registration:**

*Post hoc *analysis - clinicaltrials.gov trial NCT00106288.

## Introduction

Invasive *Candida *infections occur more often in patients housed inside rather than outside an intensive care unit (ICU) [1,2], with reported rates of candidemia ranging from 2 to almost 10 per 1,000 hospital ICU admissions. This increased incidence of invasive *Candida *infections in ICU patients is important because logistic regression analyses of data from observational studies suggest that *Candida *infection is an independent predictor of mortality among ICU patients, and both hospitalization and length of stay in an ICU are predictors of poor outcomes among patients with candidemia [3-8].

*Candida *epidemiology has changed as infections due to non-*albicans Candida *species have increased [9]. This shift in the prevalence of *Candida *species is a matter of concern because species such as *Candida glabrata *have been associated with reduced susceptibility to triazole antifungals [10-14].

The relatively high rate of infection by *Candida *spp. in ICUs, the increasing prevalence of non-*albicans Candida *spp., and the associated mortality suggest that new treatment approaches are required. One such approach may be the empirical use of antifungal agents that provide broad-spectrum coverage against *Candida *spp. [10,11,14-16]. Findings from numerous prospective and retrospective studies indicate that optimizing and reducing the delay of antifungal therapy reduces attributable mortality in patients with candidemia whereas inappropriate antifungal therapy is a significant predictor of mortality [3,17-20]. As a consequence of such findings, the most recent update to the guidelines of the Infectious Diseases Society of America includes a recommendation for the use of an echinocandin for the initial management of moderately severe to severe episodes of invasive candidiasis [14].

Micafungin is a novel echinocandin antifungal agent, which has demonstrated *in vitro *fungicidal activity against all clinically important species of *Candida *including those with resistance to fluconazole [21-27]. In two phase 3 trials, micafungin demonstrated non-inferiority to both caspofungin and liposomal amphotericin B for the treatment of invasive candidiasis and candidemia, and showed better tolerability compared with liposomal amphotericin B [28,29].

We conducted a *post hoc *analysis of the phase 3 study comparing micafungin with liposomal amphotericin B to explore the association between potential explanatory variables and clinical outcomes in adult patients who initiated antifungal chemotherapy in an ICU or in a non-ICU ward [29].

## Materials and methods

### Study objectives and design

The present study was a *post hoc *analysis of a double-blind, randomized, non-inferiority study conducted by the Micafungin Invasive Candidiasis Working Group at 115 medical centers worldwide from January 2003 to November 2004. The primary objective of this present analysis was to determine whether an ICU stay was associated with the following outcomes in patients treated for candidemia and invasive candidiasis: overall treatment success; mycological response; and all-cause mortality at day 8 and day 30 post treatment initiation. The full methodology of the study has been published previously [29]. Adult patients (age ≥ 16 years) were eligible if they had clinical signs (that is, fever, hypothermia, hypotension, local signs and symptoms of inflammation, radiologic evidence) of systemic *Candida *infection, and had one or more positive *Candida *cultures from blood or another sterile site within the previous 4 days.

Subjects were randomized to receive either micafungin (100 mg/day for patients > 40 kg; 2 mg/kg for patients ≤ 40 kg) or liposomal amphotericin B (3 mg/kg per day) as first-line treatment of candidemia and invasive candidiasis. During random assignment to their respective treatment regimens, patients were stratified by study center and neutropenia status but not by whether or not *Candida *infection developed inside or outside an ICU.

Antifungal therapy was prescribed for a minimum treatment period of 14 days and a maximum treatment period of 4 weeks - except for patients with chronic disseminated candidiasis, *Candida *osteomyelitis, or *Candida *endocarditis, for whom the study drug could be administered for up to 8 weeks. While patients with neutropenia who received antifungal prophylaxis prior to the beginning of the study were eligible, non-neutropenic patients who had received 3 days or more of systemic antifungal therapy within the previous week were ineligible. The initial doses of study drugs remained fixed during the first 5 days of treatment but a dosage increase (up to 200 mg/day for micafungin and up to 5 mg/kg/day for liposomal amphotericin B) was allowed if there was mycological persistence or ongoing clinical and radiographic evidence of infection. Conversely, a dose decrease of 50% for liposomal amphotericin B was indicated for drug-related nephrotoxicity.

Clinical and mycological assessments were made at baseline immediately prior to treatment initiation (study day 0), three times weekly during the treatment phase, and at the end of therapy. Assessments were continued at prespecified intervals post therapy for patients who were suspected of having a recurrent or emergent infection.

The study was approved by ethics committees of the participating centers, and all patients gave written, informed consent for their participation.

### Analysis population

The analysis populations consisted of all patients included in the modified intent-to-treat populations, defined as all subjects who received at least one dose of micafungin or liposomal amphotericin B and had a confirmed *Candida *infection at baseline. Subjects were retrospectively assigned to the ICU subgroup if they stayed in the ICU for at least 1 day during study days -1 to 3.

### Analysis endpoints

The analysis endpoints were as follows: overall treatment success, defined as success in both clinical response and mycological response (success in clinical response at the end of therapy defined as a complete or partial resolution of symptoms); mycological response, defined as eradication or presumed eradication of the baseline pathogen; and all-cause mortality at day 8 and day 30 post treatment initiation. A patient death during therapy was defined as treatment failure. During therapy was defined as from the date of the first dose to 1 day after the last dose.

### Statistical modeling

A series of univariate analyses were performed to evaluate associations between each treatment outcome and the ICU status. Fisher's exact test was applied for overall treatment success, mycological response, and all-cause mortality at day 8 and day 30. Potential explanatory variables (Table 1) were investigated to assess their effect on treatment outcomes. Fisher's exact test was applied if the explanatory factor was a discrete variable and the Wilcoxon rank sum test was used if the explanatory factor was a continuous variable. Explanatory variables with *P *≤ 0.1 were selected as potential confounding factors in the final multivariate models, described below as a logistic regression model.

**Table 1:** Exploratory variables used in the multivariate analyses

Class	Study value
Treatment group	Micafungin 100 mg/day
	Liposomal amphotericin B 3 mg/kg/day
Age (years)	Continuous
Sex	Female
	Male
Region	Brazil, Europe, India, North America
	Other
Race	Caucasian
	Asian-Indian
	Black
	Other
Primary diagnosis	Candidemia
	Disseminated candidiasis
Organism	*Candida albicans *only versus non-*albicans Candida*
	*C. albicans*, *Candida tropicalis*, *Candida parapsilosis*, *Candida glabrata *versus other *Candida *spp.
	*Candida parapsilosis *versus other *Candida *spp.
	*Candida krusei *versus other *Candida *spp.
Neutropenia^a ^at baseline	Yes/No
Persistent neutropenia	Yes/No
Liver disorder/failure	Yes/No
Renal disorder/failure	Yes/No
Antibiotic use	Yes/No
Diabetes mellitus	Yes/No
Solid organ transplant	Yes/No
Corticosteroid therapy	Yes/No
Catheter status^b^	Removed within 48 hours of treatment initiation, Yes/No
	Removed at any time during the study, Yes/No
Acute Physiology and Chronic Health Evaluation II score	Continuous

^a^Absolute neutrophil count < 500 cells/μl. ^b^Explanatory variable used in analysis of candidemic patients only.

The effects of ICU status on overall treatment success, mycological response, and all-cause mortality at day 8 and day 30 were evaluated using logistic regression analysis. The logistic regression model used can be described as:

where *p *is the probability of treatment success, *α *is the interception, *X *is the vector of explanatory variables, and *β *is the parameter vector to be estimated.

The ICU status and all identified potential confounding factors were included in the model as first-order explanatory variables. For each individual variable, the effect of the variable on treatment outcome in the multivariate model was tested by the Wald chi-square test. From the model, treatment outcome was compared between the levels of the variable using the odds ratio and the 95% Wald confidence interval.

## Results

### Baseline patient characteristics

Of the 537 adult subjects (age ≥ 16 years) enrolled and randomized to receive treatment with micafungin or liposomal amphotericin B, 494 subjects were included in the modified intent-to-treat analysis. Subjects were evenly distributed between the ICU (n = 263) and other hospital wards (n = 230) at the time of treatment initiation, with one subject recorded as ICU status unknown. The micafungin and liposomal amphotericin B treatment groups were well matched with respect to the proportion of assigned subjects in an ICU (48.6% versus 44.7%, respectively). Table 2 summarizes the baseline demographics and clinical characteristics of the analysis population, categorized by ICU status and treatment group.

**Table 2:** Baseline patient demographics and clinical characteristics of the modified full analysis set

Characteristic	Micafungin		Liposomal amphotericin B		Total	
	
	Non-ICU (n = 127)	ICU (n = 120)	Non-ICU (n = 136)	ICU (n = 110)	Non-ICU (n = 263)	ICU (n = 230)
Age (years)						
Mean ± standard deviation	53.1 ± 16.90	52.4 ± 19.40	53.7 ± 18.74	53.4 ± 17.76	53.4 ± 17.85	52.9 ± 18.60
Median	54.0	54.5	55.5	56.0	55.0	56.0
Range	18.0 to 87.0	18.0 to 89.0	16.0 to 89.0	17.0 to 97.0	16.0 to 89.0	17.0 to 97.0
Male, *n *(%)	79 (62.2)	76 (63.3)	79 (58.1)	68 (61.8)	158 (60.1)	144 (62.6)
Female, *n *(%)	48 (37.8)	44 (36.7)	57 (41.9)	42 (38.2)	105 (39.9)	86 (37.4)
Race, *n *(%)						
Black	11 (8.7)	2 (1.7)	7 (5.1)	3 (2.7)	18 (6.8)	5 (2.2)
Caucasian	84 (66.1)	65 (54.2)	97 (71.3)	56 (50.9)	181 (68.8)	121 (52.6)
Other	32 (25.20)	53 (44.17)	32 (23.5)	51 (46.4)	64 (24.3)	104 (45.2)
Region, *n *(%)						
Brazil	37 (29.1)	18 (15.0)	42 (30.9)	15 (13.6)	79 (30.0)	33 (14.3)
Europe	37 (29.1)	46 (38.3)	43 (31.6)	34 (30.9)	80 (30.4)	80 (34.8)
India	12 (9.4)	44 (36.7)	20 (14.7)	39 (35.5)	32 (12.2)	83 (36.1)
North America	11 (8.7)	6 (5.0)	12 (8.8)	7 (6.4)	23 (8.7)	13 (5.7)
Other	30 (23.6)	6 (5.0)	19 (14.0)	15 (13.6)	49 (18.6)	21 (9.1)
APACHE II score						
Mean ± standard deviation	13.4 ± 6.32	18.4 ± 9.39	14.1 ± 6.60	17.8 ± 9.35	13.8 ± 6.46	18.1 ± 9.35
Median	13.0	19.0	14.0	17.0	14.0	17.5
Range	0 to 30.0	0 to 44.0	0 to 37.0	0 to 47.0	0 to 37.0	0 to 47.0
Relevant risk factors						
Catheter present	95 (75.4)	116 (96.7)	93 (68.4)	105 (95.5)	188 (71.8)	221 (96.1)
Bone marrow transplant	4 (3.1)	2 (1.7)	2 (1.5)	1 (0.9)	6 (2.3)	3 (1.3)
Neutropenia	26 (20.5)	6 (5.0)	21 (15.4)	4 (3.6)	47 (17.9)	10 (4.3)
Persistent neutropenia during therapy	11 (8.8)	3 (2.5)	7 (5.3)	2 (1.9)	18 (7.0)	5 (2.2)
Acute leukemia	20 (15.7)	1 (0.8)	13 (9.6)	3 (2.7)	33 (12.5)	4 (1.7)
Hematological disorder	37 (29.1)	7 (5.8)	24 (17.6)	8 (7.3)	61 (23.2)	15 (6.5)
Liver disorder/failure	1 (0.8)	0 (0.0)	2 (1.5)	0 (0.0)	3 (1.1)	0 (0.0)
Pancreatitis	5 (3.9)	4 (3.3)	3 (2.2)	6 (5.5)	8 (3.0)	10 (4.3)
Renal disorder/failure	0 (0.0)	2 (1.7)	1 (0.7)	0 (0.0)	1 (0.4)	2 (0.9)
Solid organ tumor	23 (18.1)	12 (10.0)	27 (19.9)	23 (20.9)	50 (19.0)	35 (15.2)
Solid organ transplant	4 (3.1)	9 (7.5)	5 (3.7)	4 (3.6)	9 (3.4)	13 (5.7)
Antibiotic use	23 (18.1)	29 (24.2)	28 (20.6)	35 (31.8)	51 (19.4)	64 (27.8)
Corticosteroid therapy	14 (11.0)	22 (18.3)	20 (14.7)	17 (15.5)	34 (12.9)	39 (17.0)
Other immunosuppression	9 (7.1)	11 (9.2)	8 (5.9)	7 (6.4)	17 (6.5)	18 (7.8)
Intravenous line/device	40 (31.5)	26 (21.7)	35 (25.7)	20 (18.2)	75 (28.5)	46 (20.0)
Length of hospital stay						
Mean ± standard deviation	21.6 ± 17.77	20.0 ± 20.36	23.2 ± 20.35	27.6 ± 47.59	22.5 ± 19.13	23.6 ± 36.16
Median	18.0	14.0	19.5	15.0	19.0	14.5
Range	2 to 82.0	1 to 126.0	1 to 97.0	1 to 388.0	1 to 97.0	1 to 388.0

APACHE, Acute Physiology and Chronic Health Evaluation; ICU, intensive care unit.

While age and sex were well matched across the subgroups, there was a disparity in the racial composition of the ICU and non-ICU groups in this worldwide study. The percentage of Black subjects in the ICU group was less than that in the non-ICU group (2.2% versus 6.8%), and the prevalence of races other than Black or Caucasian were nearly twice as high in the ICU group as in the non-ICU group (45.2% versus 24.3%). There was a higher proportion of subjects from Brazil in the non-ICU group and a higher proportion of subjects from India in the ICU group.

With a few exceptions, the proportions of subjects with underlying conditions or risk factors predisposing to a nosocomial *Candida *infection were similar across the ICU and non-ICU subgroups. As expected, the mean (18.1 versus 13.8) and median (17.5 versus 14.0) Acute Physiology and Chronic Health Evaluation (APACHE) II scores were higher in the ICU group than in the non-ICU group. In addition, the presence of a central venous catheter at baseline was more frequent in the ICU group (96.1% versus 71.8%), whereas baseline neutropenia was more common in non-ICU subjects (17.9% versus 4.3%). The length of hospital stay was similar across both subgroups.

There were no substantial differences between the micafungin and liposomal amphotericin B treatment groups for ICU and non-ICU subjects. Candidemia was more common than invasive candidiasis in the present study, and the prevalence of these primary diagnoses was well matched between micafungin-treated and liposomal amphotericin B-treated subjects and across the ICU and non-ICU subgroups (Table 3).

**Table 3:** Primary diagnosis and prevalence of causative *Candida *species

Characteristic	Micafungin		Liposomal amphotericin B		Total	
	
	Non-ICU (n = 127)	ICU (n = 120)	Non-ICU (n = 136)	ICU (n = 110)	Non-ICU (n = 263)	ICU (n = 230)
Primary diagnosis						
Candidemia	109 (85.8)	98 (81.7)	115 (84.6)	94 (85.5)	224 (85.2)	192 (83.5)
Invasive candidiasis	18 (14.2)	22 (18.3)	21 (15.4)	16 (14.5)	39 (14.8)	38 (16.5)
Isolated *Candida *spp. at baseline^a^						
*Candida albicans*	44 (34.6)	58 (48.3)	55 (40.4)	54 (49.1)	99 (37.6)	112 (48.7)
*Candida parapsilosis*	29 (22.8)	13 (10.8)	22 (16.2)	16 (14.5)	51 (19.4)	29 (12.6)
*Candida tropicalis*	37 (29.1)	29 (24.2)	36 (26.5)	26 (23.6)	73 (27.8)	55 (23.9)
*Candida glabrata*	15 (11.8)	15 (12.5)	10 (7.4)	9 (8.2)	25 (9.5)	24 (10.4)
*Candida krusei*	5 (3.9)	4 (3.3)	9 (6.6)	1 (0.9)	14 (5.3)	5 (2.2)
Other *Candida *species	6 (4.7)	11 (9.2)	17 (12.5)	14 (12.7)	23 (8.7)	25 (10.9)

Data presented as *n *(%). ICU, intensive care unit. ^a^A patient may have had more than one *Candida *spp. isolated at baseline.

Overall and across the analysis groups, a non-*albicans Candida *species was more frequently isolated at baseline than *Candida albicans*. The rank order of prevalence of baseline *Candida *spp. was identical across the subgroups. Although the between-group prevalence of causative pathogens was similar, it was noted in liposomal amphotericin B-treated subjects that *Candida krusei *was isolated at baseline more frequently in non-ICU subjects (including three patients who had an underlying hematological disorder) compared with ICU subjects; nine isolates versus one isolate, respectively.

### Efficacy outcomes

Rates of overall treatment success, of mycological response, and of all-cause mortality for ICU and non-ICU subjects treated with micafungin or liposomal amphotericin B are summarized in Table 4. In non-ICU subjects, the treatment success rate was significantly higher among subjects receiving micafungin than liposomal amphotericin B (85% versus 72.1%; *P *= 0.0113). For ICU subjects, however, treatment success rates for micafungin versus liposomal amphotericin B were similar (62.5% versus 66.4%, respectively).

**Table 4:** Treatment response, mycological response, and crude mortality rates

Outcome	Micafungin		Liposomal amphotericin B		*P *value^a^		Total		
	
	Non-ICU (n = 127)	ICU (n = 120)	Non-ICU (n = 136)	ICU (n = 110)	Non-ICU	ICU	Non-ICU (n = 263)	ICU (n = 230)	*P *value
Overall treatment success	108 (85.0)	75 (62.5)	98 (72.1)	73 (66.4)	0.0113*	0.5828	206 (78.3)	148 (64.3)	0.0006*
Mycological response	109 (85.8)	88 (73.3)	106 (77.9)	79 (71.8)	0.1115	0.8825	215 (81.7)	167 (72.6)	0.2371
All-cause mortality at day 8	6 (4.7)	25 (20.8)	14 (10.3)	18 (16.4)	0.1057	0.4028	20 (7.6)	43 (18.7)	0.8935
All-cause mortality at day 30	25 (19.7)	46 (38.3)	32 (23.5)	38 (34.5)	0.4589	0.5852	57 (21.7)	84 (36.5)	0.0003*

Data presented as *n *(%). ICU, intensive care unit. ^a^Micafungin versus liposomal amphotericin B. *Statistically significant.

**Figure 1:**
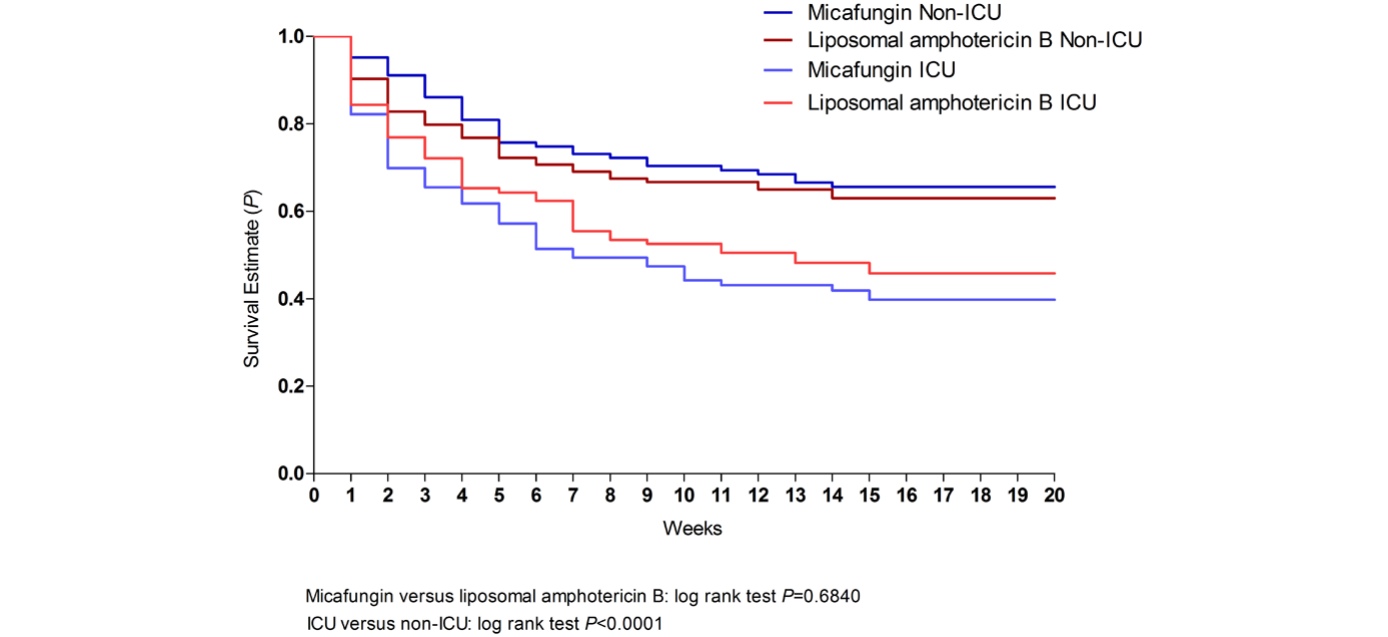
Probability of survival in subjects treated with micafungin and liposomal amphotericin B. Kaplan--Meier estimates of survival in intensive care unit (ICU) subjects and non-ICU subjects.

Rates of mycological response were slightly higher than rates of overall treatment success, and were consistent across both ICU subgroups and across each treatment group. All-cause mortality at day 8 was moderate (7.6% in non-ICU subjects and 18.7% in ICU subjects) but increased by day 30 (21.7% in non-ICU subjects and 36.5% in ICU subjects). Kaplan-Meier estimates of the probability of survival in ICU and non-ICU subjects treated with micafungin and liposomal amphotericin B are displayed in Figure 1.

When the micafungin treatment group and the liposomal amphotericin B treatment group were combined and the data analyzed only according to ICU status, the results demonstrated that fewer ICU subjects achieved overall treatment success than non-ICU subjects. This difference was demonstrated to be statistically significant (64.3% versus 78.3%; *P *= 0.0006).

### Multivariate logistic regression analyses

Multivariate regression analyses were performed in order to uncover the risk factors underlying the difference in treatment success noted in ICU subjects versus non-ICU subjects. When the logistic regression model was run without interaction terms between potential confounding factors, results revealed a lower likelihood of treatment success for ICU versus non-ICU subjects, for subjects with persistent neutropenia during therapy, and for subjects with high versus low APACHE II scores. In the logistic regression model including interactions between ICU status and potential confounding factors (where possible), however, the APACHE II score emerged as the only variable associated with each of the four prespecified outcomes analyzed (Table 5). In addition to the APACHE II score, subjects without persistent neutropenia during therapy were more likely to achieve overall treatment success even when interaction terms were included in the final analysis. Similarly, although five explanatory variables (ICU status, primary diagnosis, neutropenia at baseline, diabetes, and APACHE II score) were detected that may have influenced the mycological response on initial analysis, only the APACHE II score emerged as a statistically significant explanatory variable associated with mycological response in the final analysis.

**Table 5:** Significant predictors of overall treatment success, mycological response and mortality

Variable	Ratio	Without interaction terms^a^	With interaction terms	
			
			Maximum likelihood estimate (± standard error)	Wald χ^2 ^(probability > χ^2^)
Overall treatment success				
ICU status^b^	Not in ICU to in ICU	1.866 (1.147 to 3.034)	0.1380 ± 0.5438	0.0644 (0.7997)
Persistent neutropenia	Non-neutropenic to neutropenic	5.721 (1.412 to 23.169)	1.7185 ± 0.7156	5.7669 (0.0163)
APACHE II score	High to low (continuous)	0.956 (0.929 to 0.983)	--0.0468 ± 0.0144	10.6416 (0.011)
Mycological response				
ICU status^b^	Not in ICU to in ICU	1.778 (1.037 to 3.048)	--0.0223 ± 1.7567	0.0002 (0.9899)
Primary diagnosis	Candidemia to invasive candidiasis	2.465 (1.343 to 4.524)	0.3246 ± 0.4368	0.5522 (0.4574)
Neutropenia^b^	Non-neutropenic to neutropenic	2.357 (1.134 to 4.898)	--0.9093 ± 1.0206	3.4859 (0.0619)
Diabetes mellitus	No to yes	0.350 (0.137 to 0.894)	--1.0113 ± 0.5362	3.5572 (0.0593)
APACHE II score	High to low (continuous)	0.953 (0.925 to 0.982)	--0.0505 ± 0.0154	10.7417 (0.001)
All-cause mortality at day 8				
*Candida *spp.	*Candida krusei *to other *Candida *spp.	3.536 (1.039 to 12.035)	2.4861 ± 1.3739	3.2745 (0.0704)
APACHE II score	High to low (continuous)	1.097 (1.055 to 1.142)	0.0931 ± 0.0208	20.0498 (< 0.0001)
All-cause mortality at day 30				
Age	Low to high	1.018 (1.003 to 1.033)	0.0170 ± 0.0076	4.9331 (0.0263)
Persistent neutropenia	Non-neutropenic to neutropenic	0.160 (0.039 to 0.658)	--1.7845 ± 0.7146	6.2358 (0.0125)
APACHE II score	High to low (continuous)	1.093 (1.057 to 1.131)	0.0937 ± 0.0178	27.7797 (< 0.0001)

Significant predictors of overall treatment success, mycological response and mortality based on a logistic regression model that controlled for potential confounding variables with and without interaction terms. APACHE, Acute Physiology and Chronic Health Evaluation; ICU, intensive care unit. ^a^Odds ratio (95% confidence interval). ^b^At the time of treatment initiation.

Potential explanatory factors demonstrating an association with an increased likelihood of mortality at day 8 were *C. krusei *versus other *Candida *species and a high versus low APACHE II score. Increasing age, persistent neutropenia, and APACHE II score were associated with a higher likelihood of mortality at day 30. These associations remained statistically significant when interaction terms were included in the final model.

### Safety

Renal function was significantly better in subjects who received micafungin than those who received liposomal amphotericin B. The difference (liposomal amphotericin B group - micafungin group) in the mean peak change in the estimated glomerular filtration rate was -18.2 ml/minute/1.73 m^2 ^(*P *< 0.0001) and -17.7 ml/minute/1.73 m^2 ^(*P *= 0.0124) in non-ICU subjects and in ICU subjects, respectively.

## Discussion

Given that many ICU patients will become infected by one or more *Candida *spp. at some point during hospitalization [30], it is important that ongoing research is conducted to identify those risk factors that are most likely to influence health outcomes in this multimorbid, heterogeneous patient population.

In this *post hoc *subgroup analysis of a prospective, randomized clinical trial - conducted in line with various recommendations for *post hoc *analysis [31-35] - the rate of overall treatment success was higher in non-ICU patients receiving micafungin than those receiving liposomal amphotericin B. In ICU patients, overall treatment success rates in patients who received micafungin or liposomal amphotericin B were similar, and were lower than the corresponding treatment success rates in non-ICU patients.

Although ICU patients had lower treatment success rates than non-ICU patients, multivariate regression analysis revealed that the ICU status was not associated with treatment outcome when potential confounding factors were considered. The APACHE II score was the only potential explanatory variable associated with treatment success, mortality at day 8, and mortality at day 30. Catheter status had no effect on any outcome in patients with candidemia (data not shown).

These results seem to be at odds with *post hoc *observations from a prospective randomized study assessing the safety and efficacy of caspofungin versus amphotericin B deoxycholate in patients with invasive candidiasis [36,37]. Multivariate regression analysis indicated that patients initiating antifungal treatment in an ICU were more likely to die than those initiating antifungal therapy outside an ICU even after accounting for APACHE II score [36]. It should be noted, however, that a study of caspofungin versus amphotericin B deoxycholate treatment measured all-cause mortality 6 to 8 weeks after completion of study therapy [36] whereas the analysis we describe here measured all-cause mortality 30 days post treatment initiation.

The all-cause mortality rate at day 30 in our analysis was in general agreement with data derived from observational studies [4,38-51]. Using multivariate analyses, findings from observational studies [4,17,39,42] and a prospective clinical trial [52] have underscored the importance of the APACHE II score as a prognostic indicator. In one of the observational studies, graded APACHE II scores were not only strongly associated with 3-month mortality but a linear relationship also existed between these variables for most *Candida *spp. [39]. Furthermore, analysis of prospective, randomized, controlled trial data clearly demonstrated that the risk of failing study therapy increased incrementally with APACHE II score (odds ratio = 1.09 per points, 95% confidence interval = 1.03 to 1.14; *P *= 0.001) [52].

## Conclusions

While it is important to realize the limitations inherent in any *post hoc *analysis, the analysis described here remains one of the most extensive such investigations of the associations between the stay in an ICU and clinical outcomes in patients with confirmed candidemia or invasive candidiasis. Our findings underscore the importance of the APACHE II score as a prognostic indicator in both ICU patients and non-ICU patients with invasive *Candida *infections.

## Key messages

• In ICU patients, the overall treatment success rates in patients who received micafungin or liposomal amphotericin B were similar, and were lower than the corresponding treatment success rates in non-ICU patients.

• Renal function was significantly better in both ICU and non-ICU patients who received micafungin than in those who received liposomal amphotericin B.

• Multivariate regression analysis, along with the analysis of interactions, revealed that the relationship between the ICU status and treatment outcomes was explained by other variables. The APACHE II score was the only explanatory variable associated with treatment success, all-cause mortality at day 8, and all-cause mortality at day 30.

• These data underline the importance of the APACHE II score as a prognostic indicator of clinical outcome in patients receiving antifungal therapy in both the ICU and the non-ICU setting.

## Abbreviations

APACHE: Acute Physiology and Chronic Health Evaluation; ICU: intensive care unit.

## Competing interests

BFD has served as a consultant for Schering-Plough, Astellas Pharma, Merck, Valeant, and BioAlliance. OL has served as a speaker's bureau member for Pfizer, Astellas, Gilead Sciences, Schering Corp and MSD. LO-Z has received research grants, consulting fees, and/or speaker fees from the following companies: Astellas, Merck, Pfizer, Gilead, Sequella, and Basilea. FS is an employee of Astellas Pharma Europe BV, Leiderdorp, The Netherlands. VY has been an investigator in Astellas funded research and serves as a consultant to Astellas Pharma Inc. USA. Sponsored by Astellas Pharma Inc.

## Authors' contributions

BFD, OL, LO-Z, VY were investigators in the clinical trial on which this *post hoc *analysis is based. FS performed the statistical analysis. All authors contributed to the design of the statistical analysis and reviewed and approved the manuscript at each stage of development.
